# Clinical characteristics and management of co-infected patients of influenza A and *Aspergillus*: Case series in the Southern China

**DOI:** 10.1097/MD.0000000000043728

**Published:** 2025-08-08

**Authors:** Chongxiang Chen, Pingping Wang, Ping Peng

**Affiliations:** a Guangzhou Development District Hospital, Guangzhou Huangpu District People’s Hospital, Guangzhou, Guangdong Province, China; b State Key Laboratory of Respiratory Disease, National Clinical Research Center for Respiratory Disease, Guangzhou Institute of Respiratory Health, The First Affiliated Hospital of Guangzhou Medical University, Guangzhou, Guangdong Province, China.

**Keywords:** Aspergillus, case series, influenza

## Abstract

**Rationale:**

Influenza and *Aspergillus* co-infection is a topic of significant interest.

**Patient concerns and Diagnoses:**

We report 5 patients with influenza and *Aspergillus* co-infection of. Upon admission, all patients were diagnosed with influenza A virus infection and had elevated levels of inflammatory markers, including C-reactive protein and serum amyloid A.

**Interventions:**

The patients were treated with antiviral therapy upon admission. Computed tomography imaging 5 to 10 days later detected no improvement. Further investigation indicated that these patients exhibited signs of acute *Aspergillus* infection, showing halo signs and solidification along the bronchi. One patient was diagnosed with a co-infection by biopsy, while the remaining patients were diagnosed clinically based on at least 2 diagnostic markers (such as blood/BALF GM, tNGS, and sputum culture). After co-infection diagnosis, treatment was supplemented by the antifungal voriconazole.

**Outcomes:**

Follow-up computed tomography scans in 4 patients revealed improvement after 7 to 14 days of antifungal treatment.

**Lessons:**

It is important to note that Aspergillus infection should be considered as a potential risk factor among patients with influenza who fail to improve or worsen despite receiving full antiviral treatment.

## 1. Introduction

Influenza viruses (types A, B, and, rarely, C) primarily cause respiratory illness but can also lead to systemic complications. Severe influenza infections, especially in high-risk populations (e.g., elderly, immunocompromised, and those with chronic diseases), are often associated with complications beyond the respiratory illness, including myocarditis^[1]^ – caused by direct viral damage or immune-mediated inflammation – and invasive aspergillosis (IA), which manifests as pulmonary, disseminated,^[2–4]^ or, rarely, liver abscess forms.^[5]^ Myocarditis presents as chest pain, arrhythmias, or heart failure, requiring supportive care and antiviral medications. IA, increasingly seen in immunocompetent patients with influenza, arises from epithelial damage that facilitates *Aspergillus* invasion. Risk factors include corticosteroid therapy, intensive care unit (ICU) admission, and lymphopenia, leading to high mortality in disseminated cases. Diagnosis remains challenging and relies on imaging, cultures, and biopsies, emphasizing the need for early suspicion and antifungal therapy in high-risk individuals.

Influenza and *Aspergillus* co-infection is a topic of significant interest.^[6]^ This rare co-infection has been associated with prolonged hospitalization, increased healthcare expenses, and higher mortality rates.^[7]^ This study presents the clinical characteristics of 5 patients with influenza and *Aspergillus* co-infection, and investigates the diagnostic and therapeutic approaches employed.

## 2. Methods

This study presents patients treated with influenza at the Guangzhou Development District Hospital between May 2023 and May 2024. Influenza infection was verified by Reverse Transcription Polymerase Chain Reaction (RT-PCR) on samples collected by nasopharyngeal swabs.^[8]^ *Aspergillus* infection was identified through galactomannan (GM) in blood or bronchoalveolar lavage fluid (BALF) samples, targeted next-generation sequencing (tNGS) of BALF, sputum culture, and pathological biopsy obtained during bronchoscopy.^[9]^ The report followed the recommendations set forth in the Case Report (CARE) guidelines.^[10]^

We used VIEK 15 Compat for culture, MiNiseq DX gene sequencer for tNGS, and combined influenza A and B nucleic acid test kit for RT-PCR. All patients were diagnosed as having influenza A virus infection. Serum amyloid A (SAA) synthesized by liver cells enters the plasma as a free protein and rapidly binds to high-density lipoprotein during the acute response phase, and previous study showed that the sensitivity of SAA was 68.24% in diagnosing influenza A.^[11]^

## 3. Cases presentation

### 3.1. Case 1

**Figure 1.**
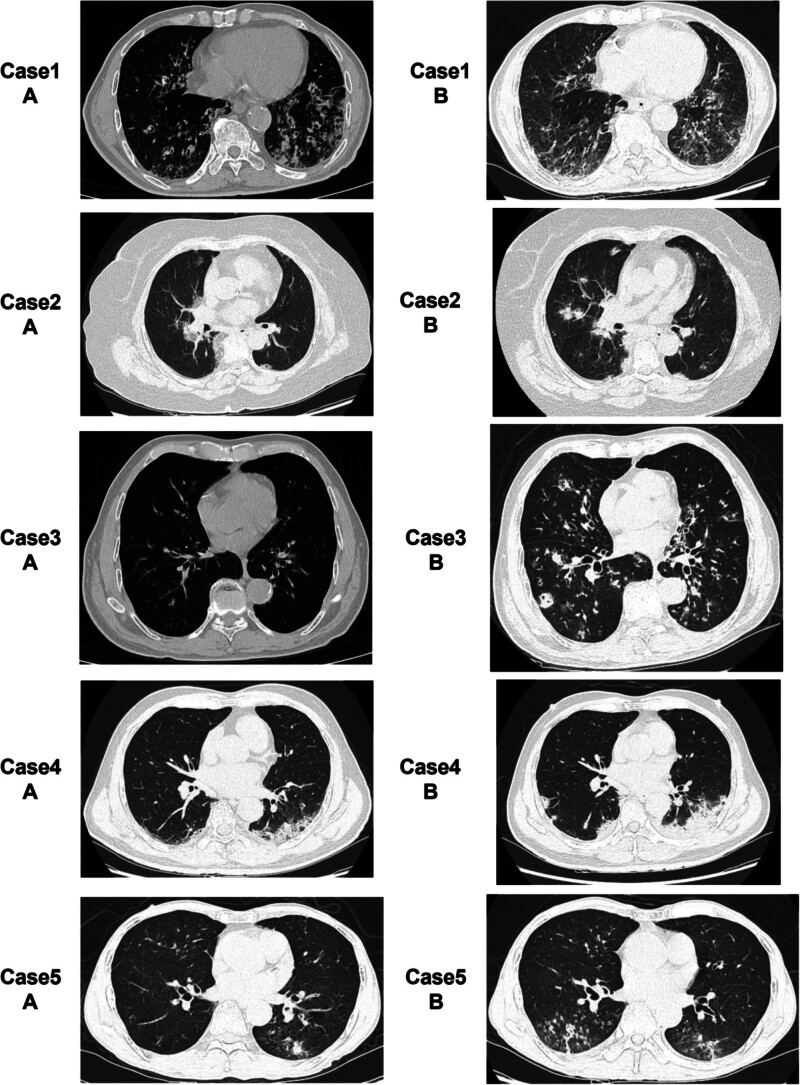
Initial (A series) and subsequent CT (B series, taken after 5–10 days of antiviral treatment and before initiating of antifungal therapy) CT images of patients with influenza and *Aspergillus* co-infection are shown. CT = computed tomography.

**Figure 2.**
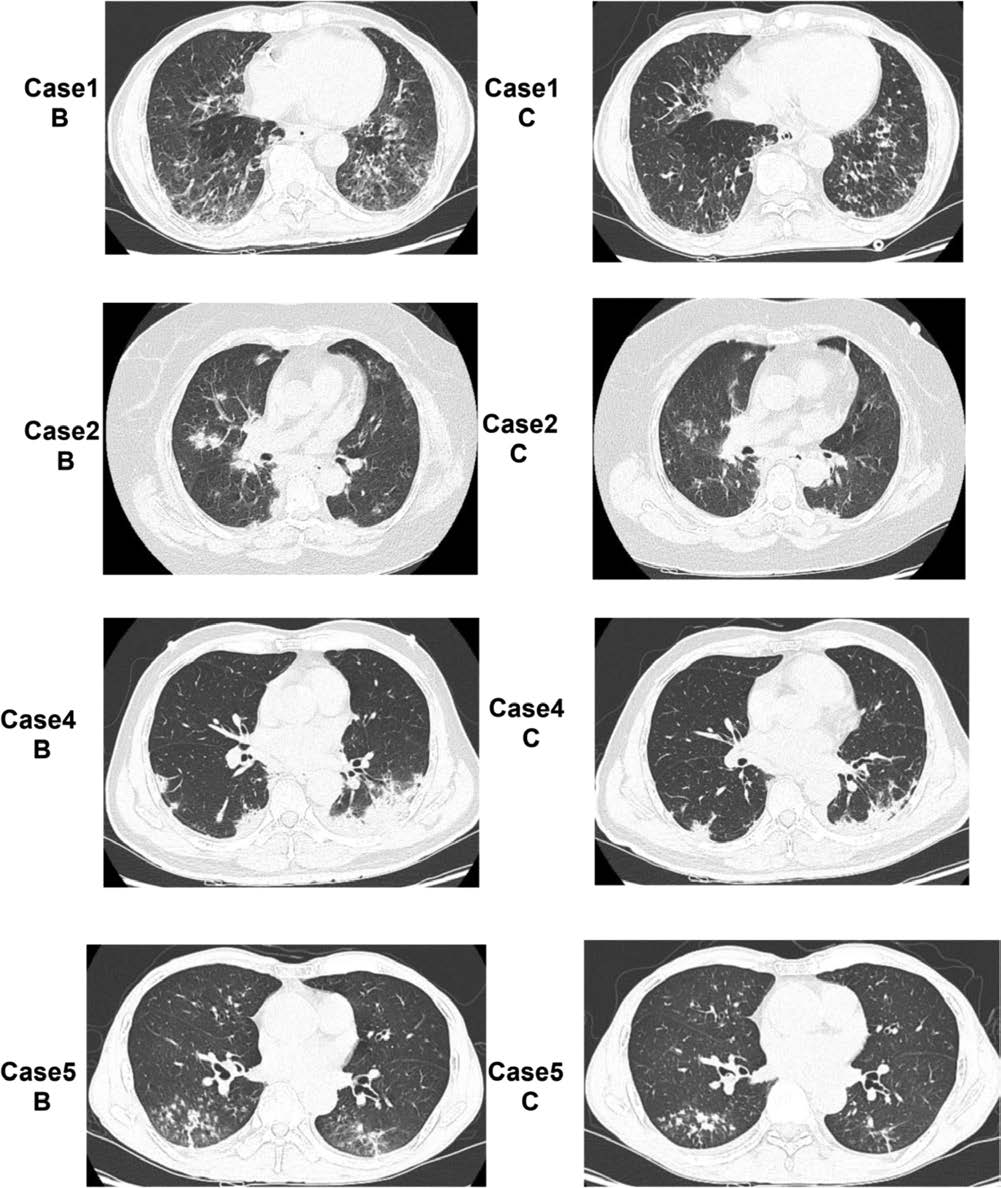
CT images were taken before initiating antifungal therapy (B series), and after 7–14 days of antifungal treatment (C series). CT = computed tomography.

A 65-year-old male with a medical history that included bronchiectasis, hypertension, diabetes, and stroke presented with acute symptoms, including coughing, fever, and dyspnea, that had lasted 5 days. influenza A virus infection was confirmed by RT-PCR. The patient received oseltamivir (75 mg, twice daily for 5 days) as antiviral treatment, but remained hospitalized for 14 days. Elevated The inflammatory markers, C-reactive protein (CRP:46.11 mg/L), SAA (320 mg/L) were elevated, while procalcitonin (0.07 ng/mL) remained normal. Follow-up computed tomography (CT) scans showed that the lung lesions observed in the initial CT scans persisted despite 5 days of antiviral therapy. BALF testing (GM and tNGS) confirmed influenza-*Aspergillus* co-infection. Four days elapsed between the influenza diagnosis and the development of IA. The patient’s therapy was supplemented with voriconazole (400 mg twice daily for 1 day, followed by 200 mg twice daily) as antifungal therapy. Radiographic improvement was noted after 9 days. The patient likely had IAPA (Tables 1 and 2; Figs. 1 and 2).

**Table 1:** The baseline characteristics of these co-infected patients with influenza and aspergillus.

No.	Gender	Age	Smoking	Drinking	Symptom	Type of influenza	Hospital stay time	Baseline disease	Antiviral treatment after admission	Prognosis (90-d mortality)
1	Male	65	No	No	Cough, fever, dyspnea	A	14 d	Bronchiectasia, Hypertension, diabetes, Stroke	Oseltamivir	Recover
2	Female	77	No	No	Cough, sputum	A	14 d	Hypertension	Oseltamivir	Recover
3	Male	75	Yes	No	Cough, fever, dyspnea	A	21 d	COPD and asthma	Oseltamivir	Death
4	Male	66	No	No	Cough, sputum, fever	A	14 d	Hypertension, diabetes	Oseltamivir	Recover
5	Male	62	Yes	No	Cough, fever	A	18 d	Chronic renal dysfunction	Mabalozavir	Recover

COPD = chronic obstructive pulmonary disease, ICU = intensive care unit.

**Table 2:** The laboratory tests of these co-infected patients with influenza and aspergillus.

No.	WBC (10^9^/L)	Neu%	Lym%	PCT (ng/mL)	CRP (mg/L)	SAA (mg/L)	GM (blood)	G test (pg/mL)	Culture (BALF/Sputum)	GM (BALF)	NGS (BALF)
1	11.22	86.10%	7.50%	0.07	46.11*	320.00*	0.40	43.94	NA	9.50*	Aspergillus fumigatus: 600:>1.0E+6
2	2.68	46.30%	38.80%	0.13	40.70*	89.31*	1.08*	376.06*	Aspergillus fumigatus	NA	NA
3	16.47	90.00%	4.20%	0.41	76.80*	280.70*	0.12	271.50*	Aspergillus fumigatus	NA	Aspergillus fumigatus: 1760:4.8E+5
4	3.30	70.30%	18.20%	0.10	66.30*	278.78*	0.18	<37.50	Aspergillus fumigatus	3.56*	Aspergillus fumigatus: 3290:<1.0E+3
5	7.1	73.10%	15.90%	0.18	48.30*	286.44*	1.20*	87.63*	NA	8.37*	Aspergillus fumigatus:13558:>1.0E+6

BALF = bronchoalveolar lavage fluid, CRP = C-reactive protein, GM = galactomannan, NA = not applicable, NGS = next-generation sequencing technology, PCT = procalcitonin, SAA = serum amyloid A, WBC = white blood cell.

* Means high.

### 3.2. Case 2

A 77-year-old woman with hypertension developed coughing with thick, yellow sputum that had lasted 8 days. Influenza A virus infection was confirmed by RT-PCR, leading to oseltamivir (75 mg twice daily for 5 days) therapy, but the patient remained hospitalized for 14 days. Inflammatory indicators were high (CRP: 40.70 mg/mL, SAA: 89.31 mg/mL) while procalcitonin (0.13 ng/mL) remained normal. Follow-up CT scans showed that the exudative changes in the lungs observed in the initial CT scans persisted despite 9 days of antiviral therapy. Blood GM and BALF/sputum culture results confirmed the dual infection. Ten days elapsed between the influenza diagnosis and the development of IA. The patient’s therapy was supplemented with voriconazole (400 mg twice daily for 1 day, followed by 200 mg, twice daily) as antifungal therapy. Radiographic imaging performed 14 days later showed notable improvement. The patient likely had IAPA. (Tables 1 and 2; Figs. 1 and 2).

### 3.3. Case 3

A 75-year-old male with a history of overlapping COPD and asthma presented with acute symptoms including cough, fever, and dyspnea, that had lasted 7 days. Influenza A virus infection was confirmed by RT-PCR. The patient received oseltamivir (75 mg twice daily for 5 days) as antiviral therapy, but remained hospitalized for 21 days and required transfer to the ICU. The inflammatory indicators CRP (76.80 mg/L) and SAA (280.70 mg/L) were elevated, but procalcitonin (0.41 ng/mL) remained normal. Follow-up CT scans showed that the slight lung abnormalities observed in the initial CT scans deteriorated despite ten days of antiviral therapy. The patient’s BALF tNGS and BALF/sputum culture results confirmed influenza-Aspergillus co-infection, 4 days elapsed between the influenza diagnosis and the development of IA. The patient’s therapy was supplemented with voriconazole (400 mg twice daily for 1 day, followed by 200 mg twice daily) as antifungal therapy. However, the patient failed to recover, requiring ICU transfer and referral to another hospital, precluding further CT evaluations. The patient likely had IAPA (Tables 1 and 2; Figs. 1 and 2).

### 3.4. Case 4

A 66-year-old male with a history of hypertension and diabetes presented with acute symptoms, including coughing, thick, yellow sputum, and fever, that had lasted 4 days. Influenza A virus infection was confirmed by RT-PCR. The patient received oseltamivir (75 mg twice daily for 5 days) as antiviral therapy, but remained hospitalized for 14 days. The inflammatory indicators CRP (66.30 mg/L) and SAA (278.78 mg/L) were elevated, while procalcitonin (0.10 ng/mL) remained normal. Follow-up CT scans persisted despite 5 days of antiviral therapy. BALF tNGS, GM, and BALF/sputum culture data established influenza-*Aspergillus* co-infection. Seven days elapsed between the influenza diagnosis and the development of IA. The patient’s therapy was supplemented with voriconazole (400 mg twice daily for 1 day, followed by 200 mg twice daily) as antifungal therapy. Radiographic imaging over the next ten days showed a notable improvement. The patient likely had IAPA (Tables 1 and 2, Figs. 1 and 2).

### 3.5. Case 5

**Figure 3.**
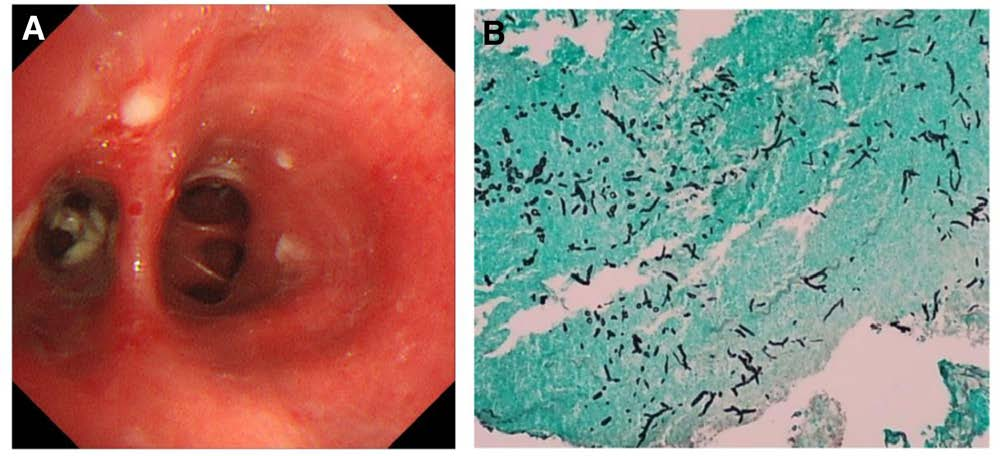
Bronchoscopy and pathological image of case 5. (A) Bronchoscopy image showing the right lower lobe basal segment in patient 5, (B) a histopathological image showing the presence of *Aspergillus* hyphae and spores in the bronchial mucosa.

A 62-year-old male with a history of chronic renal dysfunction presented with acute symptoms, including coughing, and fever, that had lasted 5 days. Influenza A virus infection was confirmed by RT-PCR. The patient received mabalozavir as antiviral therapy, but remained hospitalized for 18 days. The inflammatory markers CRP (48.30 mg/mL) and SAA (286.44 mg/mL) were elevated, while procalcitonin (0.18 ng/mL) remained normal. Follow-up CT scans showed that the exudative changes in the lungs observed in the initial CT scans persisted despite 6 days of antiviral therapy. A bronchoscopy revealed abnormal bronchial mucosa signs with sticky phlegm attached to the wall, which the patient failed to remove. This prompted a mucosa biopsy during the procedure. Pathology confirmed the presence of *Aspergillus* hyphae and spores in the bronchial mucosa (Fig. 3). Three days elapsed between the influenza diagnosis and the development of IA. The patient’s therapy was supplemented with voriconazole (200 mg twice daily for 1 day, followed by 160 mg twice daily) as antifungal therapy. Follow-up CT scans performed 9 days later demonstrated notable improvement. The patient likely had IAPA (Tables 1 and 2, Figs. 1 and 2).

## 4. Discussion

Influenza was diagnosed in 120 patients who presented at our department (general ward) between May 2023 and May 2024. Approximately 4.2% of these patients were diagnosed with influenza and *Aspergillus* co-infection. Multiple studies primarily conducted in ICUs have reported a co-infection rate of 19% and a 90-day mortality rate of 20%.^[12,13]^ And other studies discussing about influenza and *Aspergillus* co-infection, the hospital mortality was range from 18.6% to 67% (Table 3).^[12,14–18]^

**Table 3:** Previous studies discussing about the co-infection of influenza and IA.

Study	Gender (male)	ICU admission	Age	Number	Hospital mortality
Schauwvlieghe et al^[12]^	56 (67%)	ICU patients	60 (12)	83	41 (49%)
Zhao et al^[14]^	44 (44/70)	40 (57.1)	63.5 (54.3–72.8)	70	13 (18.6%)
Lee et al^[15]^	Male	NA	45	1	NA
Huang et al^[16]^	66.7%	NA	52 (±18)	18	44.4%
Lee et al^[17]^	8 (88.9%)	NA	60.98 ± 11.58	9	6 (67%)
Wu et al^[18]^	13 (54.2)	NA	67.6 14.5	24	10 (41.7)

ICU = intensive care unit.

Upon admission, all patients in our study were diagnosed with influenza A virus infection, using RT-PCR analysis. While all participants had chronic diseases, structural lung lesions were only observed in patients 1 and 3. Prolonged hospital stays (≥14 days) were required, with 1 patient requiring transfer to the ICU (Table 1). The initial CT scans, performed upon admission, are depicted in Figure 1A, while subsequent CT scans, acquired after 5 to 10 days of antiviral treatment, are shown in Figure 1B. Despite antiviral treatment, no improvement was observed in these CT findings; rather, they exhibited signs of acute Aspergillus infection, including halo signs and solidification along the bronchial direction (Fig. 1).

Four cases underwent bronchoscopy for GM and tNGS testing, revealing *Aspergillus* infection (Table 2). Voriconazole antifungal treatment was initiated in all patients once influenza and *Aspergillus* co-infections were diagnosed. Follow-up CT scans in 4 cases revealed improvement after 7 to 14 days of antifungal therapy (Fig. 2C).

One patient in our study was diagnosed by biopsy, while the remaining 4 were diagnosed clinically using at least 2 diagnostic markers (such as blood/BALF GM, tNGS, and BALF/sputum culture). Mudrakola et al^[19]^ demonstrated that diagnostic bronchoscopy, that involved biopsy and BALF, was effective in accurately identifying IA.^[20]^ The bronchial mucosa or lung tissue biopsies obtained through bronchoscopy are essential for accurate diagnosis.^[21]^ However, the timing of bronchoscopy biopsy in patients with co-infection is crucial,^[22]^ as airway lesions are a key characteristics acute Influenza and *Aspergillus* co-infection.

Blood/BALF GM is an acknowledged diagnostic biomarker for *Aspergillus* infection.^[23,24]^ Still, the importance of sputum culture in confirming *Aspergillus* infection should not be overlooked.^[25]^ However, due to its relatively low positive rate, sputum culture should only be used in conjunction with other diagnostic markers, as demonstrated in Case 2, who had positive BALF/sputum culture, blood GM, and blood G test (serum beta-D-glucan) results. Since its development, the tNGS technology has been crucial in diagnosing *Aspergillus* infection.^[24]^

Recommendations in the Infectious Diseases Society of America guidelines and other expert opinions^[26,27]^ suggest that patients with severe influenza, organ transplants, hematogenous malignancies, and immunosuppression are at higher risk of developing influenza and *Aspergillus* co-infections.^[13]^ Two of our patients exhibited decreased white blood cell counts, potentially leading to immunosuppression. Two other patients showed signs of structural lung damage (COPD and bronchiectasis), and some had chronic diseases that could predispose to co-infection, including diabetes and chronic renal dysfunction. *Aspergillus fumigatus*, an opportunistic pathogenic fungus, primarily targets the respiratory system. It penetrates the lung alveolar epithelium by destroying the cytoskeleton of alveolar epithelial cells. Consequently, multiple studies have suggested that COPD and bronchiectasis are risk factors for *Aspergillus* infection.^[28,29]^

Influenza plays a significant role in the development of *Aspergillus* infection.^[30]^ The pathogenesis of influenza-associated aspergillosis involves a combination of viral-induced epithelial damage, immune dysregulation, and impaired host defenses. Influenza A virus infects the respiratory epithelial cells, triggering extensive inflammation and destruction of the mucociliary barrier, which normally prevents fungal invasion.^[6]^ The virus also suppresses alveolar macrophage and neutrophil function, key immune defenses against *Aspergillus*. Furthermore, influenza-induced lymphopenia and the frequent use of corticosteroids in severe cases further compromise cellular immunity. These factors create a permissive environment for *Aspergillus* spores to germinate, invade blood vessels, and disseminate, leading to invasive pulmonary aspergillosis or, in severe cases, extrapulmonary manifestations such as liver abscesses and disseminated disease. Hypoxia and endothelial injury caused by severe influenza infection further exacerbate fungal tissue invasion, highlighting the critical interplay between viral infection and secondary fungal disease.^[31]^

Our study revealed that patients with co-infection exhibited elevated inflammatory markers (CRP,^[32]^ SAA^[33]^). Therefore, we propose that high inflammatory markers should be considered as a potential risk factor for Aspergillus infection in patients with influenza patients. One of our patients was admitted to the ICU and experienced a decline in health. Patients with co-infections required additional systemic support and exhibited a higher mortality rate than those with influenza infection alone.^[32]^

The CT scans of these cases did not reveal signs of chronic *Aspergillus* infection, such as the crescent sign or cavity. Instead, they displayed indicators of acute infection, including halo signs and solidification along the bronchi. Follow-up CT scans showed that the changes in the lungs observed in the initial CT scans persisted despite 5 to 10 days of antiviral therapy, requiring the patients to remain hospitalized for ≥ 14 days. The CT scans only showed improvement following 7 to 14 days of antifungal treatment, although not to the level observed in the initial scans. This finding supports the recommended 6 to 10 weeks anti-*Aspergillus* treatments.^[7]^

In our view, co-infection should be suspected in patients with severe influenza symptoms who present in an immunosuppressive state and do not improve despite about 3 days of anti-infection and anti-influenza therapy.

## 5. Conclusion

This study presented 5 cases of influenza and *Aspergillus* co-infection. *Aspergillus* infection should be considered in patients with influenza who fail to improve or worsen despite full antiviral treatment. Furthermore, individuals with structural lung damage are particularly susceptible to fungal infections. Incorporating innovative diagnostic techniques, such as tNGS, into the established guidelines is recommended.

## Acknowledgments

We acknowledge all contributed authors.

## Author contributions

**Conceptualization:** Chongxiang Chen, Pingping Wang, Ping Peng.

**Data curation:** Chongxiang Chen, Pingping Wang, Ping Peng.

**Investigation:** Chongxiang Chen, Pingping Wang, Ping Peng.

**Methodology:** Chongxiang Chen, Ping Peng.

**Writing – original draft:** Chongxiang Chen, Pingping Wang.

**Writing – review & editing:** Ping Peng.
